# Supplementary material for: Cellulose Nanocrystal Reinforced Chitosan Based UV Barrier Composite Films for Sustainable Packaging
Source: Polymers (Basel). 2020 Jan 13;12(1):202. doi: 10.3390/polym12010202 (PMC7023618; doi:10.3390/polym12010202)
Supplement: Supplementary file 1 [file polymers-12-00202-s001.pdf]

Supporting Information

Cellulose nanocrystal reinforced chitosan based UV barrier composite films for sustainable packaging

Mithilesh Yadav [1,2,*], Kartik Behera [1], Yen-Hsiang Chang [3,4], and Fang-Chyou Chiu [1,3,*]

[1] Department of Chemical and Materials Engineering, Chang Gung University, Taoyuan 333, Taiwan; b.kartik1991@gmail.com (K.B.)

[2] Department of Chemistry, Prof. Rajendra Singh Institute of Physical Sciences for study and Research, V.B.S Purvanchal University Jaunpur, U.P. 222002, India

[3] Department of General Dentistry, Chang Gung Memorial Hospital, Taoyuan 333, Taiwan; cyh4714@hotmail.com (Y.-H.C.)

[4] Graduate Institute of Dental and Craniofacial Science, Chang Gung University, Taoyuan 333, Taiwan

* Correspondence: dryadavin@gmail.com (M.Y.); maxson@mail.cgu.edu.tw (F.-C.C.); Tel.: +91-8738045471(M.Y.); +886-953678628 (F.-C.C.)

Table S1. Thermal stability of CMC, CNC, CS0, CS2, CS4, CS6, and CS8 samples.

Properties (°C and %)	Samples code						
	CMC	CNC	CS0	CS2	CS4	CS6	CS8
T_{50}[a]	322.5	324.0	307.0	309.5	301.5	306.0	302.5
T_{maxI}[a]	339.1	346.4	55.8	44.8	46.9	47.1	52.2
T_{maxII}[a]	---	---	181.0	185.2	181.2	183.1	192.8
T_{maxIII}[a]	---	---	291.2	289.8	291.6	288.9	290.0
T_{50}[b]	333.5	341.5	305.0	310.5	311.0	323.5	316.5
T_{maxI}[b]	327.1	326.8	56.5	37.8	49.0	39.0	42.3
T_{maxII}[b]	---	---	170.6	173.5	172.3	176.3	173.6
T_{maxIII}[b]	---	---	292.2	291.2	288.1	287.9	286.3
T_{maxIV}[b]	---	---	550.9	521.7	551.8	545.5	541.0
Residue (%)[a]	0.80	0.23	26.57	26.86	26.02	29.43	26.07
Residue (%)[b]	0.26	0.01	0.40	1.21	0.36	0.04	1.55

[a] in nitrogen; [b] in air.

Table S2. Comparison of mechanical properties of CS and its nanocomposites.

Source of various CNC for CS matrix	Used optimum concentration CNC (wt.%)	Thickness of CNC/CS films (mm)	Mechanical properties			References
			YM (%)	TS (%)	EB (%)*	
**Bacterial**	**4**	**0.10**	**206.45**	**96.11**	-29.80	[5]
Wood	5	0.02	87.0	25.30	-53.61	[31]
Cotton	12	0.11	161.29	5.95	-73.98	[83]
Blue gave waste	30		84	80.00	-66.67	[51]
Flax fiber	20	3.00	140.69	23.50	-58.56	[30]
Cellulose	32	0.15	100.00	25.38	-36.50	[34]
Cotton pulp	20	0.03		41.18	-21.37	[55]

Cellulose	5	0.03	24.79	20.49	-3.30	[33]
Bacterial	10	0.03	37.04	55.56	-79.41	[60]
Cellulose pulp	5	0.1	255.0	18.2	-44.20	[35]
Wood	4	0.02	78.58	39.23	-54.59	This study

*- negative value.
